# Biomarker Profile in Peripheral Blood Cells Related to Alzheimer’s Disease

**DOI:** 10.1007/s12035-025-04767-y

**Published:** 2025-03-10

**Authors:** Mirko Lomi, Filippo Geraci, Cristina Del Seppia, Cristina Dolciotti, Renata Del Carratore, Paolo Bongioanni

**Affiliations:** 1 Center Research and Innovation of Myeloproliferative Neoplasms (CRIMM), University of Florence, Azienda Ospedaliero-Universitaria Careggi, 50139 Florence, Italy; 2 Department of Medical Biotechnologies, University of Siena, 53100 Siena, Italy; 3 Institute for Informatics and Telematics, National Research Council, 56124 Pisa, Italy; 4 Institute of Clinical Physiology, National Research Council, Via Moruzzi, 1, 56124 Pisa, Italy; 5 Neuroscience Department, Azienda Ospedaliero-Universitaria Pisana, 56124 Pisa, Italy; 6 NeuroCare, OdV Onlus, 56124 Pisa, Italy

**Keywords:** PBWC, Biomarkers, Proteomic, Alzheimer’s disease

## Abstract

In a healthy brain, neuroinflammation, controlled by the main intermediary for the immune response microglia and astrocytes, contributes to maintain physiological functions such as secretion of neurotrophic factors, removal of cell tau and amyloid-β (Aβ) debris, and local homeostasis. When the immune response becomes chronic, it can become pathological and fuel neuroinflammation, causing glial cells to malfunction and not perform their function of clearing debris, resulting in further damage to neurons. Multiple studies highlight that an intense crosstalk is activated between peripheral blood white cells (PBWCs) and central nervous system (CNS). Nevertheless, how PBWC can be carriers of biomarkers of the CNS neuropathological states it is still far to be completely known. In this work, we aimed to observe how PBWC content could be related to moderate-severity of DAT in order to have early signals from of pathological neurodegeneration brain initiate. Protein analysis have been performed in PBWC of Mild Cognitive Impairment (MCI) and DAT patients in respect to those of healthy controls and differently expressed proteins have been investigated. Our data showed a deregulation of pathways involved in neurodegeneration since from MCI level and deregulated proteins that can be considered markers for DAT onset and progression.

**Supplementary Information:**

The online version contains supplementary material available at 10.1007/s12035-025-04767-y.

## Introduction

Dementia of Alzheimer’s type (DAT) is a severe and widely spread progressive neurodegenerative disorder characterized by cognitive decline and memory loss for which there are still no appropriate tests to early identify its onset [1, 2]. Current biomarkers for DAT diagnosis are mainly based on neuroimaging and cerebrospinal fluid (CSF) assessments, which however are invasive, expensive, and and not readily accessible [1]. The identification and dosage of DAT biomarkers is made complicated by the difficulty of obtaining brain biopsies, analyzing CSF, or evaluating the inflammatory state in the central nervous system (CNS) [3]. The identification of early-stage biomarkers in plasma is increasingly controversial and remains one of the most important unresolved questions in DAT research. The research of biomarkers in peripheral serum can indeed be masked by the marked heterogeneity of elements or by their low concentrations [4]. Unfolded amyloid β (Aβ) and tau proteins plaque deposits are considered the most validated markers of the initial inflammatory state in DAT brains, but their quantitative and qualitative evaluation remains controversial and not always predictive [3]. Aβ plaques and tau filaments are present in large quantities in the patients’ brain, but they can also be found in healthy individuals and no significant correlation with their serum levels has been found [5]. A coveted destination would be to identify biomarkers in peripheral blood, indicating the earliest events of inflammation in the CNS [6–8]. The role of leukocytes that migrate into the CNS during secondary neuroinflammation and their contribution to subsequent neurological disorders in inflammatory diseases remains poorly understood [9].

Several studies have reported promising data on neurodegenerative disease-biomarkers in peripheral blood immune white cells (PBWCs) that offer an alternative for earlier diagnosis and monitoring of DAT progression [10–12].

Growing evidence suggests that neuroinflammatory events caused by multiple factors at the brain level play a significant role in the development of DAT. These events activate both central and peripheral immune responses to restore the damage [13, 14] but when the immune response becomes chronically activated, it can cause even greater neuronal damage and an indirect toxic effect on neuronal degradation can be produced [9, 15].

Therefore, there is a critical need for more accessible and reliable biomarkers to identify patients with preclinical DAT. Such biomarkers may then offer a target for therapy to prevent or slow down the disease. Evaluation of the inflammatory state at CNS level through PBWC biomarkers appears to be a promising approach as they offer several advantages as potential biomarkers. PBWCs are easily extracted from the peripheral blood and can be cultured from further analysis.

In this study, we aim to investigate whether peripheral blood PBWCs can serve as a source of diagnostic biomarkers for the DAT disease to correlate with the early stages of cognitive decline. We have compared the altered proteins expressed in the PBWCs in two categories of patients different severity of cognitive deficits, namely patients with mild cognitive impairment (MCI) and patients with full-blown DAT in respect to healthy controls (CTR) in order to understand which are the most important processes involved in the onset and progression of the disease.

## Materials and Methods

### Patients Enrolled

Patients examined in this study are in charge at the department of Neuroscience-Azienda Ospedaliero-Universitaria Pisana (AOUP). We recruited subjects with MCI according to the diagnostic criteria of Petersen [16] and patients with a diagnosis of DAT with severe cognitive impairment according to the NINDS-ARDA criteria [17]. In order to verify enrollment criteria, data regarding the clinical history and pharmacologic treatments for the cognitive deficit and possible comorbidities were collected.

All subjects performed a battery of neuropsychological tests, including the Montreal Cognitive Assessment (MoCA) and the Milan Overall Dementia Assessment (MODA), which were used—together with the Activity Daily Living (ADL) and Instrumental Activity Daily Living (IADL) scales—to obtain clinical data on both cognition and functional autonomy to assign the correct Clinical Dementia Rating (CDR) scores. The CDR represents a global dementia-staging instrument primarily conceived for use in persons with DAT [18].

All subjects performed brain magnetic resonance imaging (MRI) in order to support the differential diagnoses, between degenerative and vascular dementia, according to specific cortical and subcortical changes having diagnostic significance. Particularly, reduced cortical thickness, enhanced perivascular spaces and impairment of hippocampus volume, all remarkable signs of degenerative dementia, and focal ischemic lesions as hallmarks of vascular dementia were considered. Moreover, all patients performed brain 18 F-deoxyglucose (FDG)-positron emission tomography (PET), which provides a pattern of reduced brain FDG metabolism [19].

We excluded from the study patients with a diagnosis of subcortical dementia (such as Lewy Body Disease Dementia or other neurodegenerative diseases) and demented patients with a diagnosis of vascular dementia according to the NINDS-AIREN criteria [20], or with a prevalent vascular component (i.e., “mixed dementia,” characterized by the coexistence of degenerative and cerebrovascular disease in the same demented patient) with severe psychiatric disorders, traumatic brain injury, pseudodementia, or neoplastic disease. Subjects were clinically and biochemically assessed to exclude inflammatory comorbidities.

The analysis of proteins was performed on PBWC of 18 female patients—9 MCI (CDR = 0.5) and 9 DAT (CDR = 1–2)—and 6 age (70–80 years)-matched cognitively healthy female CTR. Subjects’ diachronic clinical data such as the CDR scores and their biochemical data (106 blood analytes values) have been included in a Database. Serum, plasma, and isolated PBWC starting from whole blood are stored into the U.O. Biobank–AOUP. Each subject was registered with a code and information about the age, sex, and disease onset. Written informed consent was obtained from all the subjects and the regional Human Ethics Committee approved the study (no. 14568).

### PBWC Preparation

PBWC samples used for the analysis were prepared from peripheral blood every 4 months over a year from age- and sex-matched patients. Blood samples were diluted 1:1 with PBS, mixed carefully, and gently layered to 4 ml of lympholyte solution in a conic 15-ml tube. The samples were centrifuged at 800 × *g* for 20 min and the interphase containing total leukocytes (WBCs) carefully aspirated; 3 volumes of PBS were added to the leukocytes, centrifuged at 120 × *g* for 10 min and the supernatant removed. The cell pellets were suspended in 500 µl PBS and 20% DMSO and 80% human albumin were added. A SyLab IceCube 1810 Cd or SyLab programmed descent freezer was used for vital leukocyte cryopreservation. Leukocyte samples used include a combination of the five major types of white blood cells, i.e., basophils, eosinophils, lymphocytes (T cells, B cells, and Natural Killer cells), monocytes, and neutrophils, which number has been detected by an automated hematology analyzer. A normalization between the levels of each protein versus the number of each specific cell category could be performed. A box plot reporting the number of total lymphocyte, neutrophil, and monocyte subset population has been included in Supplementary Material Fig. S1. Morphology of PBWC samples used for the proteomic is reported in our previous work [12].

### Proteomic Analysis

Proteomics analysis was performed on PBWC by Fritz-Lipmann-Institut of Jena according to the following protocol: PBWCs were suspended in PBS and lysis buffer was added at a final concentration of 5% SDS, 100 mM HEPES, and 50 mM DTT. The samples were sonicated (Bioruptor Plus, Diagenode, Belgium) for 10 cycles (30 s ON/60 s OFF) at a high setting at 20 °C, followed by boiling at 95 °C for 7 min. Reduction was followed by alkylation with iodoacetamide (final concentration 15 mM) for 30 min at room temperature in the dark. Pellets have been washed ones in RPMI 1640 growth medium + 20% FBS and three times in DPBS, acidified with phosphoric acid (final concentration 2.5%), and 7 × S-trap binding buffer, 100 mM TEAB, 90% methanol, was added.

Samples were bound on 96-well S-trap micro plate (Protifi) and washed three times with binding buffer. Trypsin in 50 mM TEAB pH 8.5 was added to the samples (1 µg per sample) and incubated for 1 h at 47 °C. The samples were eluted in three steps with 50 mM TEAB pH 8.5, elution buffer 1 (0.2% formic acid in water), and elution buffer 2 (50% acetonitrile and 0.2% formic acid). The eluates were dried using a speed vacuum centrifuge (Eppendorf Concentrator Plus, Eppendorf AG, Germany) and stored at − 20° C.

Prior to analysis, samples were reconstituted in MS Buffer (5% acetonitrile, 95% Milli-Q water, with 0.1% formic acid) and spiked with iRT peptides (Biognosys, Switzerland). Peptides were separated in trap/elute mode using the nanoAcquity MClass Ultra-High.

Performance liquid chromatography system (Waters, Waters Corporation, Milford, MA, USA) was equipped with a trapping (nanoAcquity Symmetry C18, 5 μm, 180 μm × 20 mm) and an analytical column (nanoAcquity BEH C18, 1.7 μm, 75 μm × 250 mm). Solvent A was water and 0.1% formic acid, and solvent B was acetonitrile and 0.1% formic acid. One microliter of the sample (∼1 μg on column) was loaded with a constant flow of solvent A at 5 μl/min onto the trapping column. Trapping time was 6 min. Peptides were eluted via the analytical column with a constant flow of 0.3 μl/min. During the elution, the percentage of solvent B increased in a nonlinear fashion from 0 to 40% in 120 min. Total run time was 145 min including equilibration and conditioning. The LC was coupled to an Orbitrap Exploris 480 (Thermo Fisher Scientific, Bremen, Germany) using the Proxeon nanospray source. The peptides were introduced into the mass spectrometer via a Pico-Tip Emitter 360-μm outer diameter × 20-μm inner diameter, and 10-μm tip (New Objective) heated at 300 °C, and a spray voltage of 2.2 kV was applied. The capillary temperature was set at 300 °C. The radio frequency ion funnel was set to 30%. For data-independent acquisition (DIA) data acquisition, full scan mass spectrometry (MS) spectra with mass range 350–1650 m/z were acquired in profile mode in the Orbitrap with resolution of 120,000 FWHM. The default charge state was set to 3 + . The filling time was set at maximum of 60 ms with limitation of 3 × 10^6^ ions. DIA scans were acquired with 40 mass window segments of differing widths across the MS1 mass range. Higher collisional dissociation fragmentation (stepped normalized collision energy; 25, 27.5, and 30%) was applied and MS/MS spectra were acquired with a resolution of 30,000 FWHM with a fixed first mass of 200 m/z after accumulation of 3 × 10 6 ions or after filling time of 35 ms (whichever occurred first). Data were acquired in profile mode. For data acquisition and processing of the raw data, Xcalibur 4.3 (Thermo) and Tune version 2.0 were used. Differentially expressed proteins (DEPs), comparing MCI vs CTR, DAT vs CTR, and DAT vs MCI were produced.

### Data Analysis

Raw data were analyzed using the direct DIA pipeline in Spectronaut (v.18, Biognosysis, Switzerland) to obtain protein quantification per sample and three lists of DEPs (DAT vs CTL, DAT vs MCI and MCI vs CTL), with BGS settings besides the following parameters: protein LFQ method = QUANT 2.0, proteotypicity filter = only protein group specific, major group quantity = median peptide quantity, minor group quantity = median precursor quantity, data filtering = Qvalue, normalizing strategy = local normalization. The data were searched against a species-specific (Homo sapiens, 20.186 entries) and a contaminants (247 entries) Swissprot database. The data were searched with the following variable modifications: oxidation (M) and acetyl (Protein N-term). A maximum of 2 missed cleavages for trypsin and 5 variable modifications were allowed. The identifications were filtered to satisfy FDR of 1% on peptide and protein level. Relative quantification was performed in Spectronaut for each paired comparison using the replicate samples from each condition. The data reports were then exported and further data analyses and visualization were performed with R studio using in-house pipelines and scripts. To select differentially expressed proteins, a log2FC cutoff of 0.58 and a *q*-value 0.05 were defined to ensure the expression level within samples of the same group to be homogeneous, namely proteins with significant differences within members of the same population cannot be differentially expressed as they would not have a significant *q*-value.

Analysis was performed by FLI Core Facility Proteomics. Samples were digested in-solution, on-beads, or in-gel desalting with OASIS solid-phase extraction systems (Waters) optional: high-pH reversed-phase fractionation liquid chromatography–tandem mass spectrometry (LC–MS/MS).

Search software: Spectronaut (Biognosys) using Pulsar (Biognosys) [21] MaxQuant Freeware [22] using Andromeda Freeware [23] Mascot server Matrix Science [24]. Data analysis and visualization: in-house developed procedures build on R\Bioconductor Spectronaut (Biognosys) Proteome Discoverer (Thermo)SpectroDive (Biognosys) SpectroMine (Biognosys) Skyline (MacCoss Lab) [25].

### PCA Analysis

The variability between samples was represent by a principal component analysis (PCA) plot. In proteomics, matrix reduction is based on the amount of protein calculated with Spectronaut. The 2-D PCA plot realized using the factoextra library within the R (version 4.3.0) framework shows the two principal components (PC1 and PC2; therefore, the first two axes that maximize the explained variance of the system) and how the samples are distributed considering these two coordinates.

### Volcano and Venn Analysis

The Volcano plot provides a graphical representation of the candidate proteins to be biomarkers, reporting on the *x*-axis the log2 Fold Change (LFC) and on the *y*-axis the Log (logarithm in base 10) of the adjusted *p*-value, expressed as *Q*-value. The thresholds to define upregulated (red dots) and downregulated (blue dots) proteins are as follows: 0.05 for the *Q*-value (or FDR) and ± 0.58 for the LFC. For the 50 most up- and downregulated proteins were indicated the names. DIA approach was described [21] through the Spectronaut software. Quantitative analyses were performed through MaxQuant [Cox 2008] using Andromeda [23]. Venn plots were produced using the Venn-Diagram library. All other figures were obtained using R's core functions.

### Gene Set Enrichment Analysis

Proteins resulted differentially expressed in each group’s comparison DAT vs CTR; MCI vs CTR and DAT vs MCI were used to perform a Gene Set Enrichment Analysis (GSEA) through DAVID Functional Annotation Bioinformatics Microarray Analysis online tool (https://david.ncifcrf.gov/tools.jsp). The GSEA analysis considers differentially expressed DEPs within the range of LFC ≥ 0.58 and LFC ≤ −0.58. The protein accession number was submitted and our analysis focused on Gene Ontology (Biological Process, Cellular Component and Molecular Function), Kyoto Encyclopedia of Genes and Genomes (KEGG), and Reactome. Terms with a False Discovery Rate (FDR) < 0.05 were selected. Dot plots, in which the enriched terms are represented, were realized under the R studio development framework (R version 4.3.0) using the ggplot2 library.

### Protein–Protein Interaction Meta-analysis

Starting from DEPs from each pairwise comparison, a protein–protein interaction (PPI) meta-analysis was performed by STRING database (https://string-db.org/). Protein lists were submitted with default settings and interactome figures were exported. Statistical analysis was performed by Bonferroni variation. The differences were considered significant at a value of *p* ≤ 0.017.

## Results

### Characteristics of the Study Participants

With the aim of identifying biomarkers relating to the initial stages of the disease, we compared the DEPs between MCI patients who show the first symptoms of cognitive impairment with DAT who are in an advanced stage of dementia. The analysis of proteins was performed on PBWC of 18 patients: 9 MCI (CDR 0, 5), 9 DAT (CDR 1–2), and 6 cognitively normal CTR.

### Protein Description

**Fig. 1:**
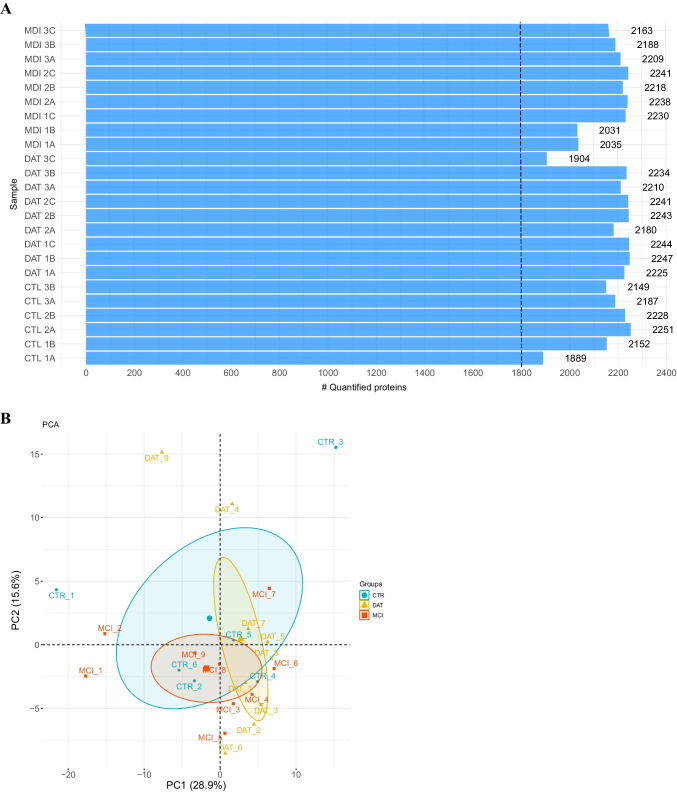
Protein quantification for 9 MCI, 9 DAT, and 6 CTR, represented by bar graph (**A**). All samples passed the quality control, as the number of proteins identified for each subject is homogeneous and is beyond the threshold value at 1800, dotted line. PCA analysis (**B**) spatial separation and clustering (within ellipses) of 9 MCI or 9 DAT patients and 6 controls (CTR) based on the change in abundance of their proteomic data

Number and quantification of DEPs are reported for each sample (Fig. 1A). All samples passed the quality control, as the number of proteins identified for each subject is beyond the threshold line (1800). A total of 2257 DEPs were detected, and details are reported in supplementary material (Table S1) PCA analysis represent how MCI and DAT patients and controls clustered by principal components related to protein content and amount (Fig. 1B). It is possible to observe the groupings of similar subjects within the ellipses.

Once the quality and clustering of the proteins has been assessed, the DEPs of the two groups of patients MCI and DAT were compared with each other and with CTR group.

### Differential Protein Expression of PBWC

**Fig. 2:**
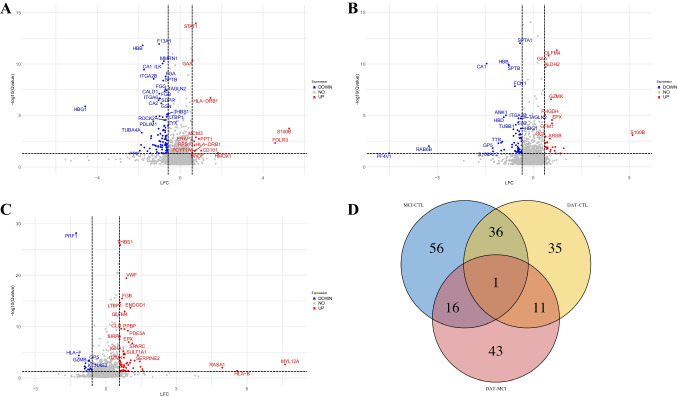
Volcano plot representing the DEPs obtained from the comparison between MCI vs CTR (**A**), DAT vs CTR (**B**), and DAT vs MCI (**C**). Data points in red represent upregulated and blue represent down-expressed proteins. Venn diagram of DEPs identified in the different severity categories of Alzheimer disease comparison (**D**). A complete list of DEPs is reported in Table S1

The comparison between MCI vs CTR highlights 414 DEPs with FDR < 0.05 (Fig. 2A), 109 of which included in the range LFC ≥ 0.58 and ≤ − 0.58. A complete list of DEPs is reported in Table S1.

Among the 109 proteins, 14 were overexpressed in MCI, while the other 95 were down-expressed. The comparison between DAT vs CTR revealed 370 DEPs with FDR < 0.05, 83 of which are included in the range LFC ≥ 0.58 and ≤ − 0.58. Among these proteins, 26 are overexpressed and 57 are down-expressed (Fig. 2B). The comparison DAT vs MCI revealed 421 statistically significant proteins with FDR < 0.05, 71 of which included in the range LFC ≥ 0.58 and ≤ −0.58. Among these, 55 are overexpressed and 16 down-expressed (Fig. 2C). DEPs among MCI, DAT, and CTR were represented in a Venn diagram (Fig. 2D). The numbers included in each section are referred to the DEPs, the names of which are reported in Table S2. Total DEPs were 83 in DAT vs CTR, 109 in MCI vs CTR, and 71 in DAT vs MCI (Table 1).

**Table 1:** List of the up- or downregulated proteins detected for each comparison: the detailed description of each DEPs abbreviations is reported in Table S1

DAT vs CTR		MCI vs CTR			DAT vs MCI	
Upregulated	Downregulate	Upregulated	Downregulate		Upregulated	Downregulate
OLFM4	SPTA1	STAT1	TLN1	ARHGAP18	F13A1	PRF1
GAA	CA1	GAA	VCL	RALB	THBS1	HLA-F
ALDH2	HBB	HLA-DRB1	F13A1	ITGB1	VWF	GP5
GZMK	SPTB	S100B	HBB	CD36	FGB	KCNAB2
PHGDH	FCN1	MCM3	MMRN1	CORO1C	LTBP1	GZMB
EPX	ANK1	PPT1	ILK	MYLK	ENDOD1	AHSG
GFM1	HBD	ERAP2	CA1	INF2	OLFM4	NFATC2
S100B	ITGA2B	FOLR3	FGA	SRC	CLU	ERAP2
GCA	TAGLN2	RPS10	ITGA2B	CYFIP1	PPBP	CTSD
ARSB	CA2	CD101	SPTB	GP1BA	PDE5A	GCDH
SPI1	TUBB1	PCYT1A	FGG	EMILIN1	SIRPA	PCK2
PPT1	HBG1	HMOX1	TAGLN2	PSTPIP2	EPX	LHPP
ERP29	BLVRB	MYOF	FGB	GDI1	SPARC	CES1
HEXA	ZYX		CALD1	ITGA2	IGHA1	TMX3
PCYT1A	PRF1		SDPR	SLC2A3	GLB1	HMOX2
RNASE2	HSPB1		ITGA6	LRBA	ACADS	NUCKS1
PDIA5	FYB		CA2	ALOX12	SULT1A1	
MX2	TMX3		GSN	CSRP1	GZMK	
NIPSNAP3A	BIN2		HBG1	ENDOD1	SERPINE2	
GSTM2	CALD1		THBS1	LRRFIP2	PROS1	
TRAP1	PDLIM1		LTBP1	PPBP	MAOB	
CRIP2	ROCK2		HBD	GRHPR	PLXDC2	
CD177	ITGA6		ANK1	NEXN	GCA	
MCCC2	SLC4A1		WDR44	TTR	SERPINE1	
HLA-DRB1	HLA-F		TUBB1	RAB27B	HPCAL1	
CD101	TUBA4A		ZYX	FKBP1A	SYTL4	
	PFKP		ROCK2	MAPRE2	TCN1	
	TTR		PDLIM1	LIMS1	HLA-E	
	RSU1		STXBP2	TPM1	MYL12A	
	ACTR3		PLEK	SPARC	VNN2	
	HMGN1		TPM4	SNTB1	DMTN	
	MAPRE1		PARVB	ASAP1	CLC	
	RAB6B		SULT1A1	PPIF	GSTM2	
	SNCA		EHD3	CD9	LIMS1	
	EMILIN1		BIN2	CDC42	RNASE2	
	CORO1C		HPSE	KALRN	SERPINH1	
	GP5		BLVRB	ASAP2	GFM1	
	CSRP1		TUBA4A	PTPN11	ARSB	
	GZMH		PFKP	GP6	VAMP7	
	CD36		SNCA	DCTN2	RASA1	
	ARHGAP18		RSU1	DMTN	RNASE3	
	MAPK1		STX11	LGALSL	APOE	
	FKBP1A		SYTL4	UQCRFS1	ITGA2	
	PPIA		VWF	KTN1	MCU	
	ROCK1		PDE5A	CNST	GNAZ	
	KIF5B		CTTN	TUBA8	CD226	
	MYO18A		GP5	PROS1	PRG2	
	S100A12		TJP2		DNAJC3	
	CDC42				HLA-B	
	FHOD1				FKBP15	
	UBE2V1				ANO6	
	PTK2B				CYP27A1	
	LRRFIP2				SLC9A9	
	CCS				APPL2	
	PF4V1				IGHM	
	UBA7					

### Gene Set Enrichment Analysis and Protein–Protein Interaction

**Fig. 3:**
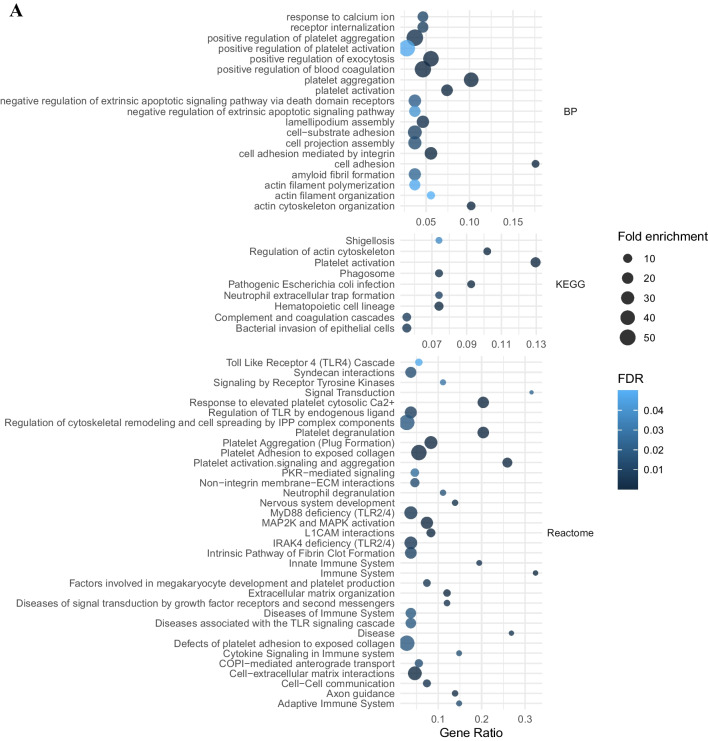

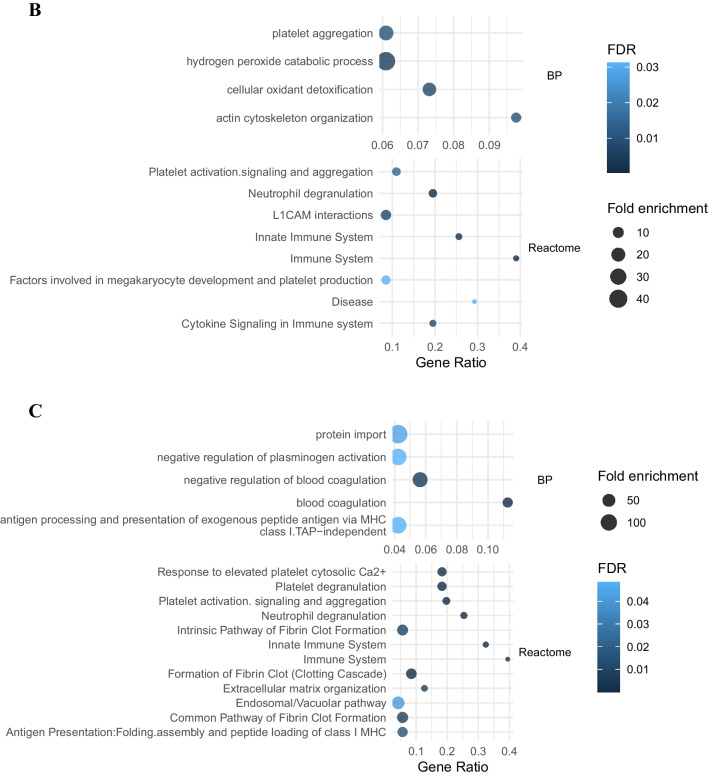
GSEA. Main biological pathway enrichment analysis based on Gene Ontology (GO) database, identified by DEPs outcomes between MCI vs CTR (**A**), DAT vs CTR (**B**), and DAT vs MCI (**C**). Terms found statistically significant by Reactome, KEGG, or GO BP between the different patients’ categories. “Gene ratio” refers to the percentage of total candidate genes in the given pathway. The number of proteins involved in each pathway (Count) fold enrichment (as dot size) and the statistical significance, as false discovery rate (FDR), are reported

DEPs have been analyzed by GSEA through databases which provide bioinformatics tools for the visualization and interpretation of pathways involved at pathophysiological level in the disease onset and severity. Three research tools, i.e., Gene Ontology (BP, CC and MF), KEGG, and Reactome, were considered for all evaluations to find statistically significant processes (FDR ≤ 0.05) related to the disease. In the following graphical representation, we have chosen to describe some of the most significant ones such as those relating to the activation of the immune system or to the crosstalk of leukocytes with the brain’s immune system such as neutrophils (Fig. 3A).

GSEA of DEPs derived from the comparison between MCI vs CTR leads to the identification of 182 processes included 93 GO terms (36 BP, 38 CC, and 19 MF), 16 KEGG terms, and 73 terms from Reactome (all detailed differentially expressed terms are reported in Table S2).

### GSEA of MCI vs CTR

The proteins found significantly different in MCI are involved in the main pathways inflammation, immune system activation innate (FDR = 2.15 × 10–8), adaptive response (FDR = 3.3 × 10–2), neuronal processes, immune response, and neutrophil degranulation [28], supposed to be induced at the DAT onset.

**Fig. 4:**
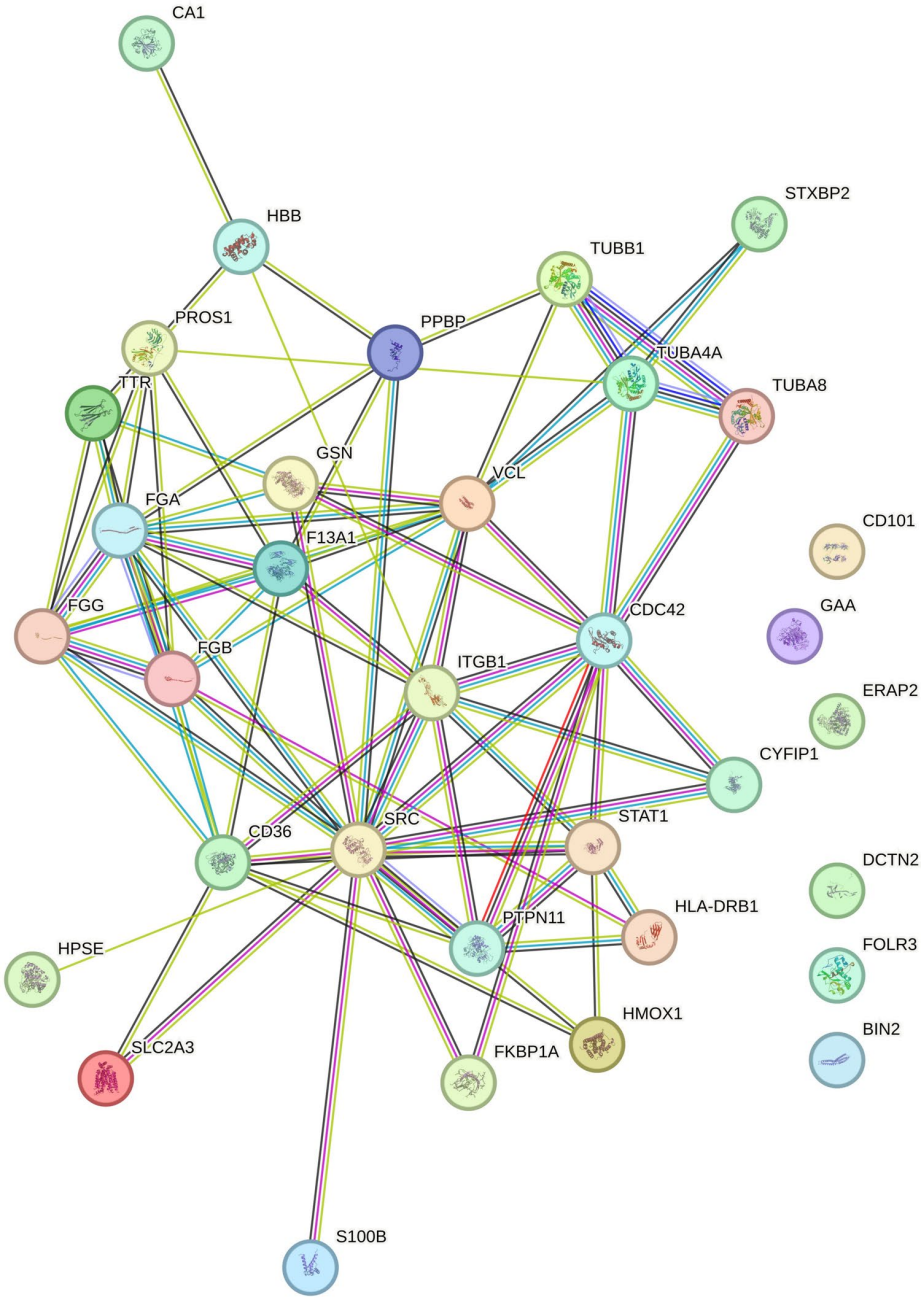
PPI obtained by DEPs between MCI vs CTR analyzed by STRING interactome tool. Proteins’ interconnection representing “Immune System” process is shown. The circles indicate the proteins, and lanes indicate the number of interactions. The circles with structure in them are proteins with known or predicted structure. Colors represent the confidence score of a functional association

A protein–protein interaction (PPI) has been performed. PPI involved in the immune system process (28 proteins) are shown in Fig. 4. All interactions are shown in supplementary (Fig. S2). PPI analysis of DEPs obtained from the comparison between MCI vs CTR reports activated proteins with a central role in immune system such as FGA, FGB, FGG, F13A1, GSN, VCL, CDC42, ITGB1, CD36, SRC, STAT1, CYFIP1, PTPN11, and HLA-DRB1 (Fig. 4). The consistent number of DEPs in this process indicates that they have an important involvement in the initial phase of progression from a more severe DAT state (Fig. 4).

The comparison of DEPs between DAT and CONT by GSEA leads to the identification of 35 GO elements (6 in BP, 18 in CC, and 11 in MF) and 13 terms in Reactome MF (Table S2). The innate component of the immune system (FDR = 1.03 × 10^−3^) includes 21 of the DEPs in DAT vs CON. Among these, we saw that there are 8 overexpressed proteins (CD177, S100B, ARSB, EPX, GCA, GAA, OLFM4, and RNASE2) and 13 down-expressed (CD36, ROCK1, S100A12, ACTR3, BIN2, CDC42, FCN1, HBB, MAPK1, PPIA, TTR, UBE2V1, and UBA7).

**Fig. 5:**
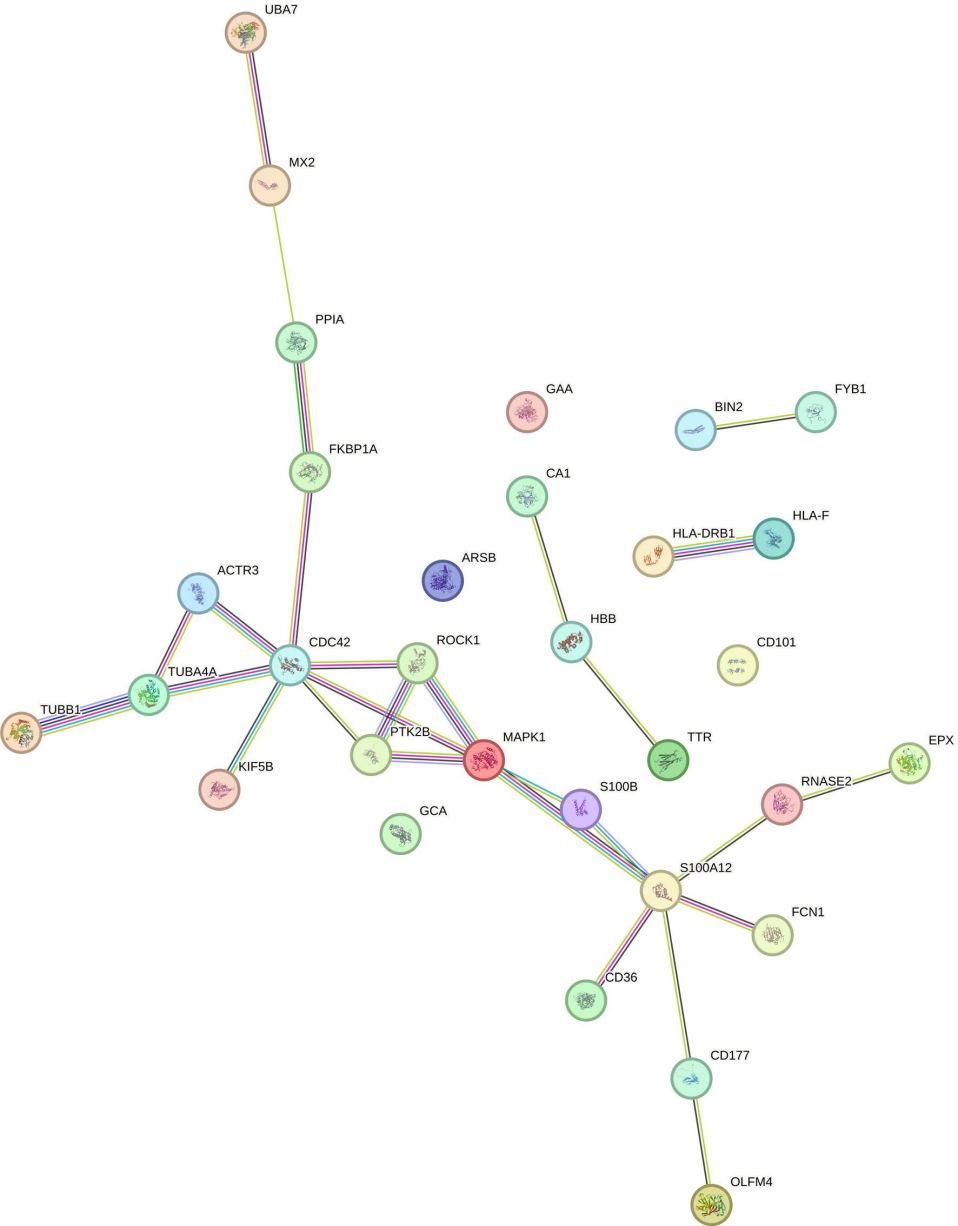
Protein–protein interaction (PPI) analysis on DEPs between DAT vs CTR. This highlights a big cluster that contains proteins which we suppose have a central role in immune system process in DAT patients. These proteins are described in the text. Analysis was performed by STRING Interactomic tool

One interesting, activated process in DAT is the binding between proteins (“protein binding” FDR = 2.24 × 10–3) (Table S2), to which 71 of the 83 proteins that were DEPs participate in this function. A further significant element “tau protein binding” (FDR = 1.77 × 10–2) known to be characteristic of DAT together with Aβ. This function involves 4 proteins, ROCK1, ROCK2, S100B, and SNCA. The neutrophil degranulation includes 16 differentially expressed proteins, among which 7 were found overexpressed (CD177, GAA, ARSB, EPX, GCA, OLFM4, and RNASE2) and 9 down-expressed (CD36, ROCK1, S100A12, BIN2, FCN1, HBB, MAPK1, PPIA, and TTR) in DAT. The comparison of DAT vs controls shows the two main clusters that most diversify advanced forms of dementia Fig. 5. All interactions are shown in the supplementary material (Fig. S3).

**Fig. 6:**
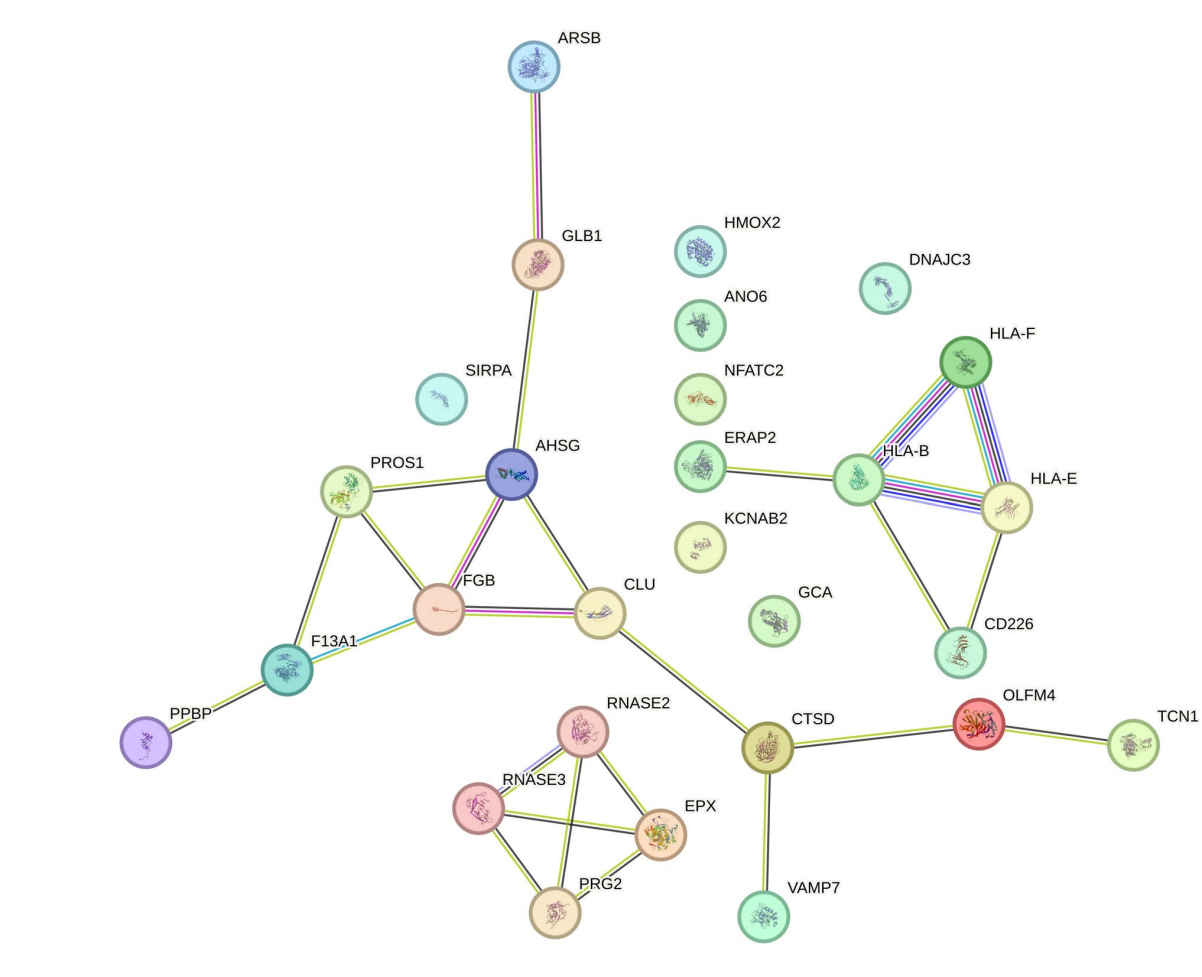
Protein–protein interaction (PPI) GSEA analysis of DEPs between DAT vs MCI that belong to immune system process. Proteins are classified in three clusters with few interactions. Analysis was performed by STRING tool

GSEA analysis of DEPs between DAT and MCI leads to the identification of 71 proteins. Twenty-five terms are highlighted as enriched in GO (of which 5 are from BP, 19 from CC, and 1 from MF), 6 terms are in KEGG, and 13 in Reactome (Table S2). Proteins that interact among themselves involved in immune system process (FDR = 1.43 × 10–4) (28 proteins) are shown in Fig. 6. All interactions are shown in the supplementary material (Fig. S4). Total PPI biological processes are reported in Table S2 and also in this comparison, elements involved in the immune system processes are particularly represented (FDR = 1.43 × 10–4). The main pathways found to be statistically significant by Reactome analysis are related to the immune system and to neutrophil degranulation process. The neutrophil degranulation includes 16 differentially expressed proteins, among which 7 were found overexpressed (CD177, GAA, ARSB, EPX, GCA, OLFM4, and RNASE2) and 9 down-expressed (CD36, ROCK1, S100A12, BIN2, FCN1, HBB, MAPK1, PPIA, and TTR) in DAT.

PPI of DEPs between DAT vs MCI highlight three groups of connected proteins indicating the processes in which these proteins are involved are crucial in the shift from MCI to severe dementia (Fig. 6). Our results indicate that, in PBWC at peripheral level, there is a general alteration of the immune responses in the phase preceding DAT.

## Discussion

PBWCs share many pathological features usually found in the CNS thus coming to represent a promising model for brain diseases. Nevertheless, papers that report specific biomarkers related to neurodegenerative pathological alterations in blood are still sporadic and discordant [5, 10, 11, 26]. In PBWC, the presence of proteins and pathways deregulated in MCI and DAT patients and involved specifically in disease onset mechanisms as well could increase elements related to the Alzheimer signatures.

In this work, we focused on the involvement of PBWCs as carriers of early markers of neuronal damage in MCI and DAT patients. We have firstly identified DEPs comprised in pathways typically related to Alzheimer such as Aβ fibril formation, tau protein binding process [3, 4] or neuronal cell adhesion, L1-CAM, or GAA, a potential protein for the treatment of DAT by virtue of its ability to clear Aβ inducing autophagosomes [27]. More importantly, we observed in MCI the activation of pathways related to neuroinflammation (i.e., neutrophil and platelet degranulation, extracellular trap formation, microglial pathogen activation, chemokine-related pathways) and to the immune system both innate and adaptive (i.e., microglia activation, interferon-induced proteins) [28].

Neuroinflammatory events always more considered the main cause associated to DAT onset [9] initiated in response to a variety of stimuli such as toxic metabolites oxidative stress, or viral or bacterial infection [29], which can promote both the central and peripheral immune response mediated by PBWC to restore the damage. One explanation of how PBWC might be a mirror of CNS is that once they reach inflamed brain compartments, they initiate crosstalk with immune systems (glia etc.), to alert the entire organism on the presence of a problem [2, 6, 30, 31]. When immune response became chronic, it goes to feed the neuroinflammation in an anomalous way, contributing to neurodegeneration and Alzheimer pathogenesis progression [15]. PBWC could be modified by the contact with microglia the main brain damage sensor years before the onset of the full-blown disease [32]. Thereafter, PBWCs, in particular neutrophils, engage in reverse transendothelial migration, returning to the bloodstream [6]. Moreover, various pathological states are known to cause disturbances to the BBB, and its permeability is affected in, e.g., brain injury, multiple sclerosis, Parkinson’s disease, and Alzheimer’s disease [33, 34]. Increased BBB permeability can facilitate the transendothelial migration and peripheral inflammatory profile may be more reflective of the CSF. PBWCs are involved both in the first intervention in the areas of neuroinflammation of the CNS probably contributing to neurodegenerative processes and then as peripheral transporters of information.

Among single proteins upregulated in DAT, there is Olfactomedine (OLFM4), transported by neutrophils whose role is still controversial [35]. OLFM4 is an important regulator of apoptosis and at a peripheral level it can be a marker of infection. Granzyme K (GZMK) is predominantly expressed by innate-like lymphocytes and is upregulated with immune activation [36]. It is a strongly connected to CD8T2 cytotoxicity including perforin 1 and mitochondrial genes. Grancalcin (GCA), a bone marrow-derived protein in immune cells, competitively binds to the low-density lipoprotein receptor-related in microglia, inhibiting phagocytosis and clearance of Aβ and potentiating neuropathological changes which negatively correlate with cognitive function [37]. G Elongation Factor Mitochondrial 1 (GFM**1**) is involved in the regulation of normal mitochondrial function but its role in diseases attributed to mitochondrial dysfunction is not known [38]. Eosinophil protein X (EPX), also known as eosinophil-derived neurotoxin, is a basic cellular protein with potent cytotoxic and neurotoxic properties and is a marker of eosinophil activation and degranulation [39]. During immune response, eosinophils are activated (turned on), and they travel to the area of injury or inflammation to release proteins and other compounds with a toxic effect on severely damaged cells or invading organisms.

Of particular interest is the presence of S100B, a cytosolic calcium-binding protein member of the S100 protein family mediating the crosstalk between the innate and adaptive immune system [40]. It is concentrated in astrocytes in the nervous system and therefore is released following astroglial injury, astrocytosis, and neurite proliferation [41]. In various physiological and pathological conditions, S100B might function as an interface to immunological processes, lymphocytes, which are unique human peripheral blood lymphocytes (PBL) containing the S100B protein reported to be involved in the pathogenesis of DAT even if little is still known about its role [40]. S100B levels in biological fluids are recognized as a reliable biomarker of active neural distress, and more recently, growing evidence indicate S100B as a damage-associated molecular pattern [40], designating it as a potential early marker of the DAT in blood. Another interesting protein upregulated in MCI and DAT in respect to controls is SPARC (secreted protein acidic and cysteine rich) produced by microglia, highly expressed in DAT brain and collocated toward Aβ protein deposits [42]. SPARC converts anti-inflammatory macrophages into a pro-inflammatory phenotype with induction of interferon-stimulated gene (ISG) expression via the transcription factors IRF3/7. Mechanistically, SPARC-induced ISGs were dependent on toll-like receptor-4 (TLR4)-mediated TBK1, IRF3, IFN-β, and STAT1 signaling without engaging the Myd88 pathway [43, 44]. The matricellular protein SPARC induces inflammatory interferon response in macrophages during aging [43], actively contributing to cerebral inflammation and subsequent tissue repair. Interdicting heightened SPARC protein expression may confer a novel therapeutic opportunity for modulating AD progression [42]. Leukocyte‐derived SPARC acts at later stages to resolve inflammation; hence, the cell specificity, temporal actions of SPARC, and how they affect the disease process certainly require further investigation. The role of SPARC in the immune response is less well understood and is expressed by various immune cell types, including macrophages, follicular dendritic cells, and CD4 + T cells. Indeed, loss of SPARC has been associated with enhanced activation of immune and inflammatory responses in a number of experimental models [44]. Thus, SPARC may serve as a potential “biomarker” for targeted therapies in certain patients and disease settings, but this is an area of increasing interest and investigation.

Several members of the serpine family, serpine1, 2, and HI, have been found induced in DAT. Serpins represent the most broadly distributed superfamily of protease inhibitors known as stress-associated endoplasmic reticulum protein. Serpins might play a role in the pathophysiology of neurodegenerative disorders, particularly by taking part in protein misfolding and in the regulation of neuroinflammation. They contribute to a variety of physiological functions and any alteration of the serpin-protease equilibrium can lead to severe consequences. Serpine dysregulation has been associated with early-stage DAT and prion diseases [45]. Not many studies have focused on serpin isoforms and their relevance in DAT. Therefore, a deeper understanding on serpine occurrence may suggest new therapeutic strategies for neurodegenerative disorders.

While the differential expression of S100B in MCI and DAT patients compared to that of healthy controls clearly highlights the role of S100B as a diagnostic molecular marker for cognitive impairment, SPARC behavior, given its protective effects on cell viability, represents a sort of therapeutic marker, overexpressed in DAT, compared to MCI patients, just as it would represent a brain attempt (with reverberation in the peripheral blood) to protect or save cell nerves from neuroinflammatory damage. All these proteins could be considered potential biomarkers associated with DAT.

## Conclusions

We found that PBWCs represent a good model to indicate the early events preceding DAT and it will be very interesting to understand the origins for this sharing to promote understanding of how peripheral leukocytes during disease progression might be informative and predictive of DAT for diagnostic purposes. Altogether, our results could contribute to establish an early signature for DAT. Our most important challenge now is to confirm and increase a strong group of biomarkers which can help diagnosis and early therapies. Data from PBWC can be used to subdivide patients based on the specific molecular signature, thus representing a reliable procedure for patient classification.

## Supplementary Information

Below is the link to the electronic supplementary material.

Supplementary file1 (PDF 19 KB)

Supplementary file2 (PDF 5.42 MB)

Supplementary file3 (PDF 3.08 MB)

Supplementary file4 (PDF 2.10 MB)

Supplementary file5 (XLSX 95 KB)

Supplementary file6 (XLSX 100 KB)

## Data Availability

No datasets were generated or analysed during the current study.

## Abbreviations

CDR: Clinical Dementia Rating
CNS: Central nervous system
CSF: Cerebrospinal fluid
CTR: Control
DAT: Dementia of Alzheimer’s type
DEPs: Differentially expressed proteins
GSEA: Gene Set Enrichment Analysis
MCI: Very mild severity of dementia
PBWC: Peripheral blood white cells
PCA: Principal component analysis
PPI: Protein-protein interaction
