# Supplementary figures and images for: Biomarker Profile in Peripheral Blood Cells Related to Alzheimer’s Disease
Source: Mol Neurobiol. 2025 Mar 10;62(7):8949–64. doi: 10.1007/s12035-025-04767-y (PMC12208969; doi:10.1007/s12035-025-04767-y)

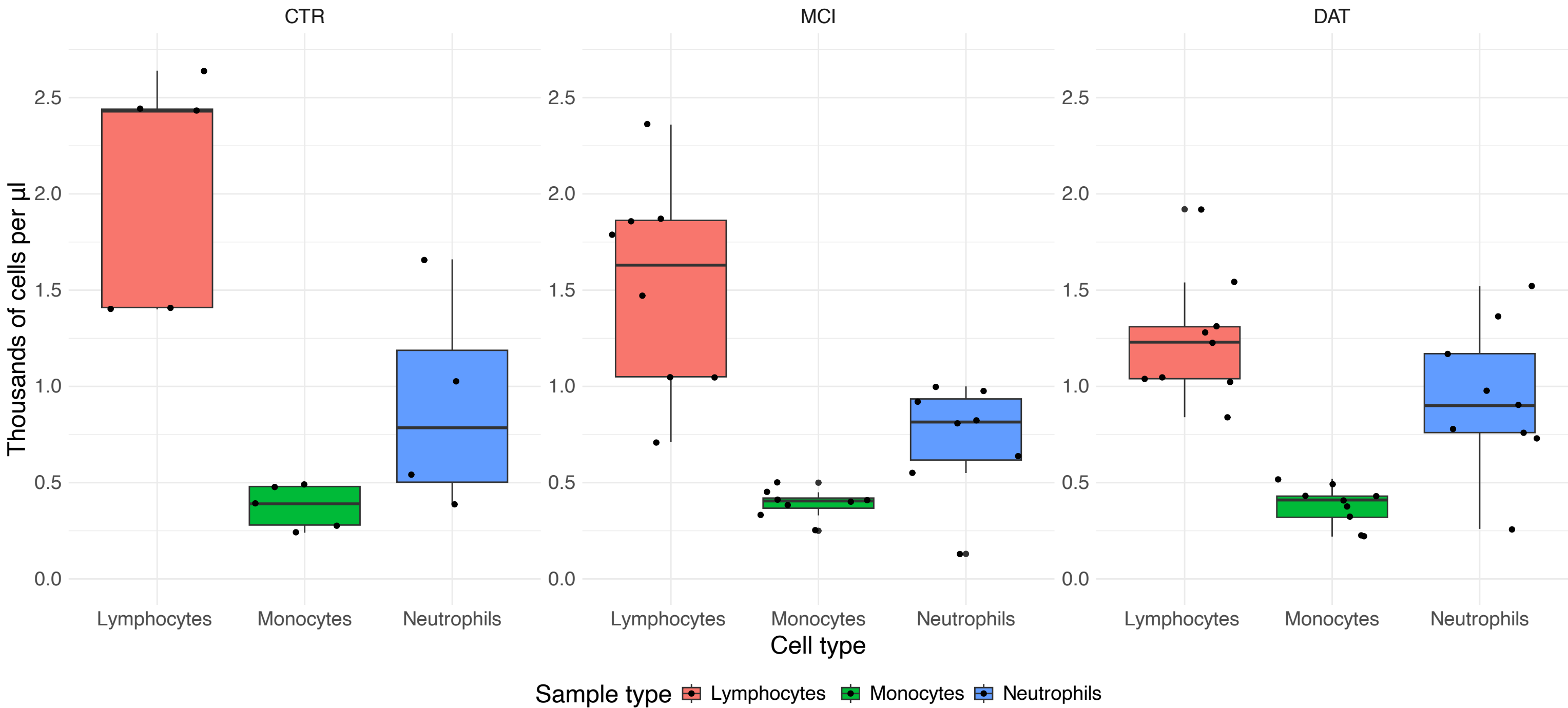

Supplement: Supplementary file 1 — Supplementary file1 (PDF 19 KB) [file 12035_2025_4767_MOESM1_ESM.pdf]

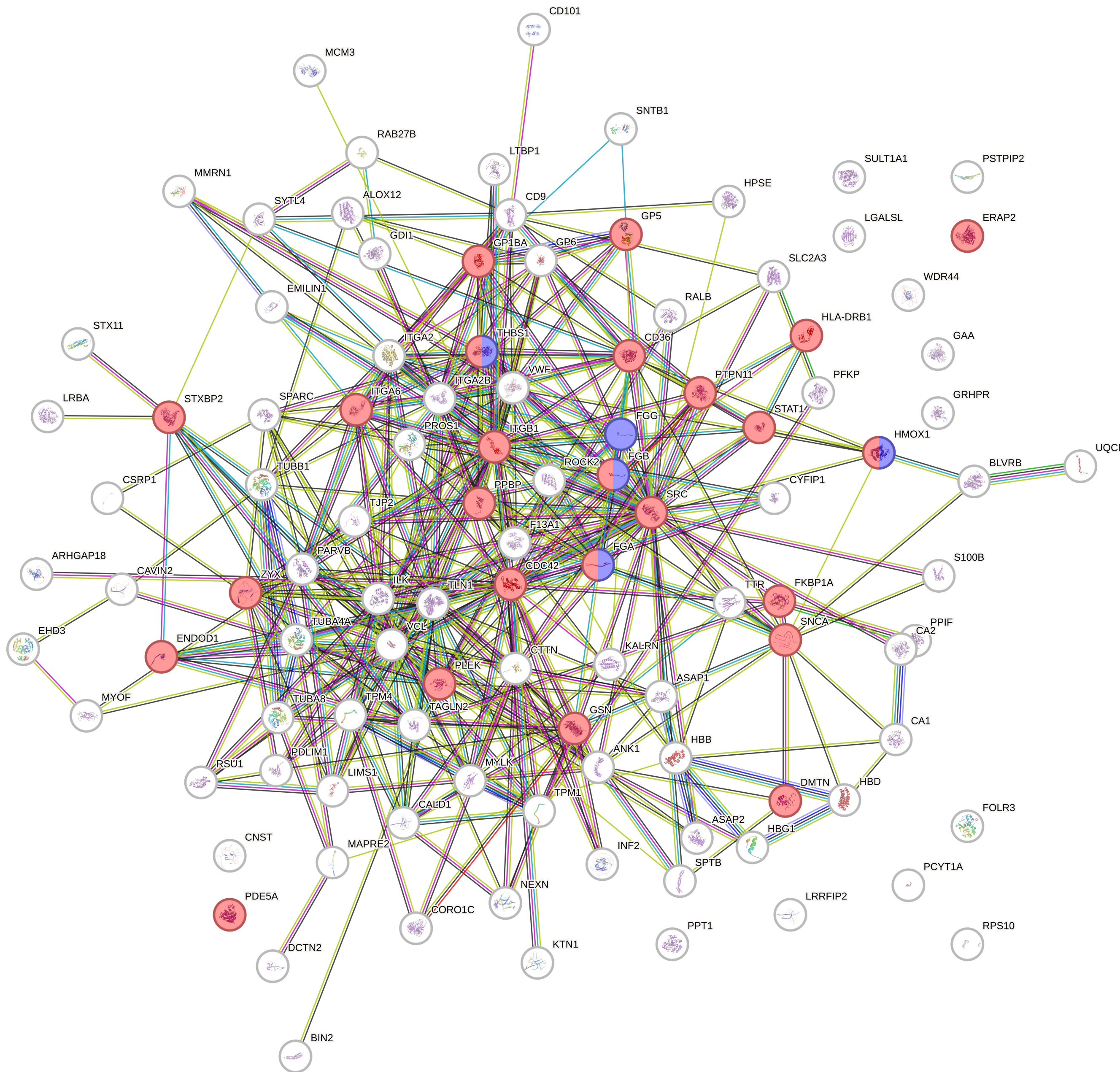

Supplement: Supplementary file 2 — Supplementary file2 (PDF 5.42 MB) [file 12035_2025_4767_MOESM2_ESM.pdf]

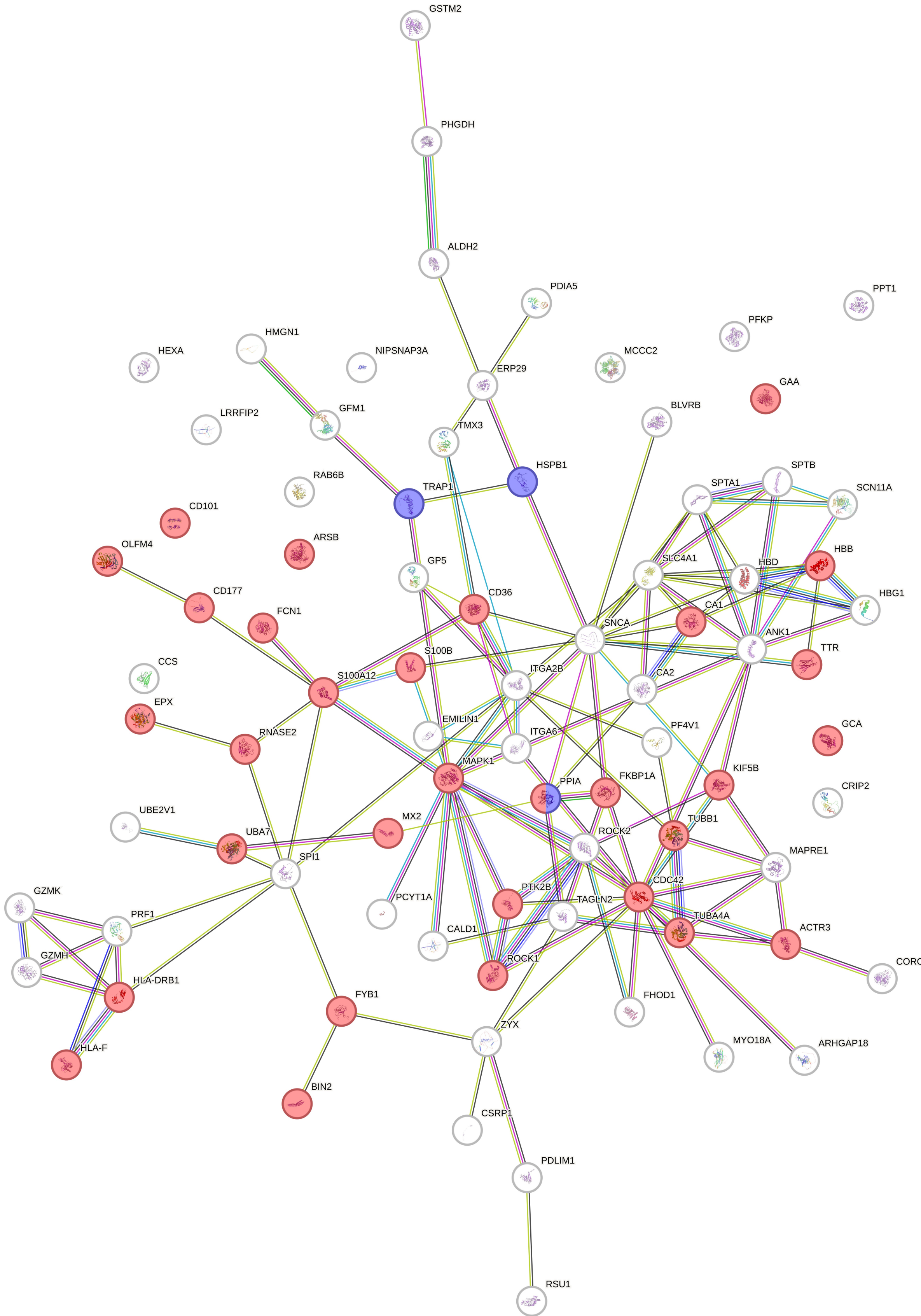

Supplement: Supplementary file 3 — Supplementary file3 (PDF 3.08 MB) [file 12035_2025_4767_MOESM3_ESM.pdf]

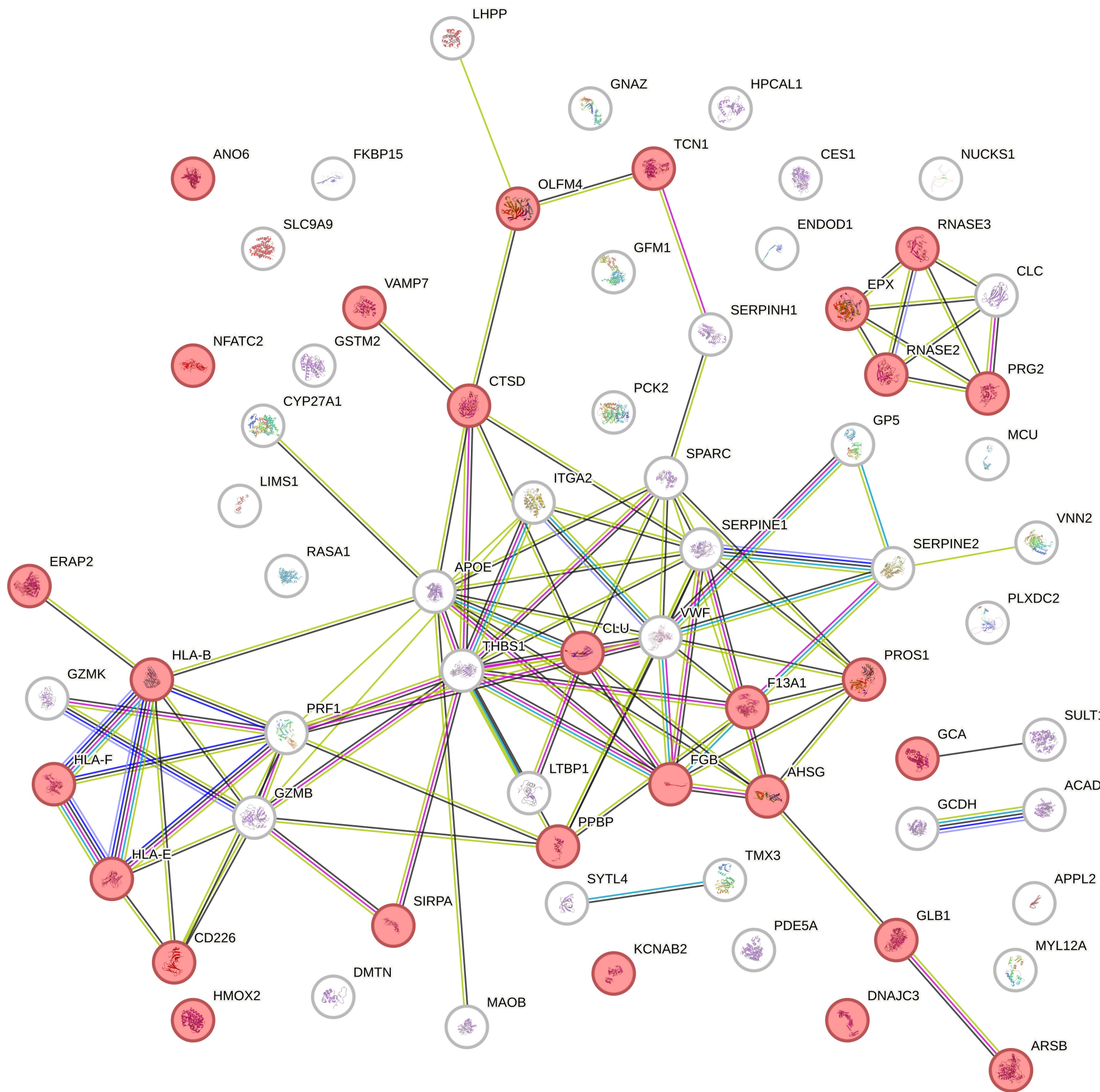

Supplement: Supplementary file 4 — Supplementary file4 (PDF 2.10 MB) [file 12035_2025_4767_MOESM4_ESM.pdf]
